# Commentary: Risk factors for complications in elderly patients aged 85 years and over undergoing endoscopic biliary stone removal

**DOI:** 10.3389/fsurg.2023.1139745

**Published:** 2023-02-28

**Authors:** Paolo Mazzola, Valentina Spedale

**Affiliations:** ^1^ School of Medicine and Surgery, Università Degli Studi di Milano-Bicocca, Monza, MB, Italy; ^2^ Acute Geriatrics Unit, Fondazione IRCCS San Gerardo dei Tintori, Monza, MB, Italy; ^3^ Nurse Educator Undergraduate Nursing Degree, Fondazione IRCCS San Gerardo dei Tintori, Monza, MB, Italy

**Keywords:** Biliary stone removal, elderly, ercp, endoscopic retrograde cholangiopancreatography, risk factors, endoscopy

A Commentary on Risk factors for complications in elderly patients aged 85 years and over undergoing endoscopic biliary stone removal By Zhang DY, Zhai YQ, Zhang GJ, Chen SX, Wu L, Chen DX, et al. (2022) Front. Surg. 9:989061. doi: 10.3389/fsurg.2022.989061

## Introduction

We read with interest the paper by Zhang and colleagues (1) that explored the risk factors for complications among patients aged ≥85 years who underwent endoscopic retrograde cholangiopancreatography (ERCP) for biliary stone removal. We recently investigated a similar issue among subjects aged ≥65 years undergoing ERCP in an Italian hospital (2). Italy is experiencing a progressive and significant aging of its population (3), and potentially an increasing number of biliary stone cases and ERCP procedures as observed in many other countries. A recent multi-center Italian study on quality in ERCP showed that the procedures met overall good quality standards, with 93.1% success rate and 8.0% complication rate (4). However, this prospective study did not consider the age of subjects undergoing ERCPs.

In 2018, our research group explored the safety of ERCP among geriatric subjects consecutively admitted to the Endoscopy Unit during a 2-year period (2). We compared two age groups (65–79 years vs. ≥80 years) in terms of complications, and we found that the younger group had a higher rate of ERCP-related complications than the older one (13.2% vs. 10.0%, respectively) as well as a significantly higher Charlson Comorbidity Index (CCI) score.

## Findings

According to Zhang et al. (1), we show the characteristics of patients aged ≥85 years (*N* = 90, see Table 1). We found a mean age of 88.8 ± 3.2 years, with a prevalence of females (72.2%) and a median CCI of 1 (Interquartile range: 0.2). Interestingly, 71.1% of the population had a low comorbidity burden (CCI 0–1), while only 9 subjects had a relevant comorbidity burden (CCI ≥ 4). Most subjects were classified with the American Society of Anesthesiologist (ASA) physical status score as class II (“mild systemic disease,” 57.8%) or III (“severe systemic disease,” 40.0%). Urgent ERCPs were performed in 11 cases (12.2%). We observed a lower comorbidity burden in this group than in the younger group (65–79 years old), and similarly the complication rate was lower among subjects ≥85 (8.9%). As expected from the low number of cases (*n* = 8), the univariate logistic regression analyses for the potential risk factors of complications in this age group did not show any significant association.

**Table 1:** Characteristics of patients aged 85 years and older undergoing ERCP, and list of their ERCP-related complications.

Characteristics	Age ≥85 years (*N* = 90)
Mean age, years ± SD	88.8 ± 3.2
Gender, *n* (%)	
Male	25 (27.8)
Female	65 (72.2)
Charlson Comorbidity Index (CCI), median (IQR)	1 (0,2)
CCI 0-1	64 (71.1)
CCI 2-3	17 (18.9)
CCI ≥ 4	9 (10.0)
ASA physical status classification score, class	
I	–
II	52 (57.8)
III	36 (40.0)
IV	2 (2.2)
V	–
Urgent ERCP, *n* (%)	11 (12.2)
Indication	
CBD stones/ biliary colic	36 (40.0)
Stenosis	13 (14.4)
Acute Pancreatitis	5 (5.6)
Stent placement or removal	18 (20.0)
Cholangitis	15 (16.7)
Other	3 (3.3)
Procedures	
Sphincterotomy	22 (24.4)
Stent placement or removal	30 (33.3)
Sphincterotomy and stent placement	27 (30.0)
Other	4 (4.4)
Incomplete procedure	7 (7.8)
Diagnosis	
CBD stones	39 (43.3)
Stenosis	14 (15.6)
Stent placement or removal	26 (28.9)
Other	4 (4.4)
Complications, number of cases	
Septic cholangitis	2
Cardiopulmonary	2
Bleeding	0
Acute Pancreatitis	2
Perforation	1
ERCP-Related death	1
Total number of complications (%)	8 (8.9)

Data are presented as absolute number and percentage, i.e. *n* (%), unless otherwise specified.

CBD, common bile duct; IQR, interquartile range; SD, standard deviation.

## Discussion

We interpreted our findings considering a possible selection bias, i.e., the lack of a standardized comprehensive geriatric assessment (CGA) for elderly subjects accessing endoscopy service. Although the CCI is a validated and user-friendly index, we agree with Zhang et al. (1) that it cannot be the only tool considered because it cannot capture alone the clinical complexity of geriatric patients. In fact, although they may possess the criteria to undergo ERCP, elderly patients might be erroneously excluded from this procedure because of their comorbidity burden. Moreover, the ASA score itself is a classification system that mostly relies on clinical judgment since it does not encompass an absolute measure of the severity of the acute and chronic comorbid conditions. Our study also had limitations: the small sample size, the single-center design, and its retrospective nature that do not allow us to generalize the findings. Conversely, one of our strengths was the presence of a younger control group.

As for present and future research aimed at improving the preoperative evaluation of geriatric subjects, we speculate that adding the assessment of frailty using validated tools might play a role in identifying patients at higher risk of ERCP-related complications. To date, very limited research about frailty in this setting is currently available. For example, a case report by Occhipinti et al. (5) described a 70-year-old man admitted to the Emergency Department with a diagnosis of acute pancreatitis in the presence of multiple gallbladder stones who underwent ERCP. The authors define him as “frail,” but this judgment is based on clinical conditions and comorbidities rather than on specific frailty assessment.

We think that Zhang et al. (1) significantly contributed to enlighten the potential risk factors of ERCP-related complications in the over 85 population, paving the path for further research in an age group characterized by complex health needs and a high burden of comorbidity. Nevertheless, we suggest including the assessment of frailty in the preoperative evaluation because of its prognostic significance in the geriatric population.

## References

[1] Zhang DY Zhai YQ Zhang GJ Chen SX Wu L Chen DX Risk factors for complications in elderly patients aged 85 years and over undergoing endoscopic biliary stone removal. Front Surg. (2022) 9:989061. 10.3389/fsurg.2022.989061 36303850 PMC9592906

[2] Galeazzi M Mazzola P Valcarcel B Bellelli G Dinelli M Pasinetti GM Endoscopic retrograde cholangiopancreatography in the elderly: results of a retrospective study and a geriatricians’ point of view. BMC Gastroenterol. (2018) 18(1):38. 10.1186/s12876-018-0764-4 29540171 PMC5853060

[3] Mazzola P Rimoldi SM Rossi P Noale M Rea F Facchini C Aging in Italy: the need for new welfare strategies in an old country. Gerontologist. (2016) 56(3):383–90. 10.1093/geront/gnv152 26553737

[4] Donato G Occhipinti P Correale L Spadaccini M Repici A Anderloni A A prospective study on quality in endoscopic retrograde cholangiopancreatography (ERCP): trend in Italy from the request study. Endosc Int Open. (2021) 9(10):E1563–71. 10.1055/a-1531-4691 34540552 PMC8445684

[5] Occhipinti V Segato S Carrara A Orlando S Conte D. Ercp or no ercp: the case report of a frail patient. Intern Emerg Med. (2018) 13(3):367–71. 10.1007/s11739-017-1732-7 28875255

